# Effects of diaphragmatic myofascial release on gastroesophageal reflux disease: a preliminary randomized controlled trial

**DOI:** 10.1038/s41598-019-43799-y

**Published:** 2019-05-13

**Authors:** I. Martínez-Hurtado, M. D. Arguisuelas, P. Almela-Notari, X. Cortés, A. Barrasa-Shaw, J. C. Campos-González, J. F. Lisón

**Affiliations:** 1 Department of Physiotherapy, Universidad Cardenal Herrera-CEU, CEU Universities, Valencia, Spain; 2 Department of Gastroenterology, Hospital General de Castellón, Castellón, Spain; 3 Department of Medicine, Universidad Cardenal Herrera-CEU, CEU Universities, Valencia, Spain; 4 Digestive Disease Department, Hospital of Sagunto, Valencia, Spain; 5 Department of Surgery, Universidad Cardenal Herrera-CEU, CEU Universities, Valencia, Spain; 6 Hospitales Vithas Nisa Virgen del Consuelo & 9 de Octubre, Valencia, Spain; 7 Hospital Arnau de Vilanova, Valencia, Spain; 8 CIBER of Physiopathology of Obesity and Nutrition CIBERobn, CB06/03 Carlos III Health Institute, Madrid, Spain

**Keywords:** Gastrointestinal diseases, Therapeutics

## Abstract

The purpose of this study is to investigate whether implementing a myofascial release (MFR) protocol designed to restore the myofascial properties of the diaphragm has any effect on the symptoms, quality of life, and consumption of proton pump inhibitors (PPI) drugs by patients with non-erosive gastroesophageal reflux disease (GERD). We randomized 30 patients with GERD into a MFR group or a sham group. Changes in symptomatology and quality of life were measured with the Reflux Disease Questionnaire and the Gastrointestinal Quality of Life Index. Need of PPIs was measured as the milligrams of drug intake over the 7 days prior to each assessment. All variables were assessed at baseline, one week and 4 weeks after the end of the treatment. At week 4, patients receiving MFR showed significant improvements in symptomatology (mean difference-1.1; 95% CI: −1.7 to −0.5), gastrointestinal quality of life (mean difference 18.1; 95% CI: 4.8 to 31.5), and PPIs use (mean difference-97 mg; 95% CI: −162 to −32), compared to the sham group. These preliminary findings indicate that the application of the MFR protocol we used in this study decreased the symptoms and PPIs usage and increased the quality of life of patients with non-erosive GERD up to four weeks after the end of the treatment.

## Introduction

Gastroesophageal reflux disease (GERD) is a condition which develops when reflux of stomach contents causes troublesome symptoms or complications^1^. This is a common burden whose prevalence is around 20% in the western world and is increasing globally^2^. Current standard treatment consists of lifestyle modifications or the administration of proton pump inhibitors (PPIs); surgery is the final option when these treatments fail^3^. However, long term consumption of PPIs has recently been related to some important side effects^4^.

In the search of an alternative treatment to the use of PPIs in patients with non-erosive GERD, some studies have demonstrated that performing periodic respiratory exercises aimed at strengthening the crura of the diaphragm (CD) is an effective non-pharmacological treatment that increases patient quality of life, decreases the perception of symptoms, and the need for PPIs^3,5–7^. The idea of exercising the diaphragm is based on the assumption that the CD is a key component of the antireflux barrier because it functions as an extrinsic esophagogastric junction (EGJ) sphincter^8^.

Among the many manual therapy techniques available, myofascial release (MFR) techniques are widely-used. MFR treatments require the application of three-dimensional low-load pressures to the fascial tissue over extended periods with the aim of manipulating the myofascial complex and restoring its optimal length. These treatments have been shown to reduce pain and improve the function of the treated areas^9–12^. By applying either a MFR technique designed to stretch the diaphragm muscle fibres or a sham technique in a group of patients with GERD while performing high-resolution oesophageal manometry, Da Silva *et al*. (2013) showed that the pressure exerted by the lower oesophageal sphincter (LES) immediately increased in patients treated with MFR but in not those in the sham group^13^.

To our knowledge, so far, no studies have evaluated the efficacy of MFR in improving GERD symptoms. Thus, the purpose of this preliminary study was to investigate the effects of a MFR protocol designed to restore the myofascial properties of the CD.

## Method

### Design

This study was designed as a parallel, sham-controlled trial with balanced randomisation; it followed the Consort recommendations for clinical trials^14^. The trial was conducted following the ethical requirements established in the 1964 Declaration of Helsinki and its sixth revision in 2008^15^, and was approved by the Universidad Cardenal Herrera Human Ethics Committee. All the participants read and signed the informed consent statement and gave informed consent for the publication of identifying information/images in an online open-access publication before being included in the study. The trial was conducted between October 2017 and March 2018, and was registered at ClinicalTrials.gov (NCT03299985, 3/10/2017).

### Participants, therapists, centres

Patients were enrolled from different medical centres and included adults (aged 18 to 80 years) with a physician’s diagnosis of GERD according to the Montreal definition^1^ and who had undergone a previous endoscopy study. Exclusion criteria were: endoscopically proven hiatal hernia or current erosive esophagitis and previous surgery at the LES. Other exclusion criteria were: congenital or acquired immune disorders, an allergic status of any kind, systemic diseases (rheumatic, infectious conditions, febrile state, vascular alterations, endocrine diseases including diabetes, metabolic, and neoplastic syndromes), leukaemia, severe psychiatric disorders, neuromuscular or neurological injuries, aneurysms, abdominal or spinal surgery, vertebral fractures, advanced-stage osteoporosis, acute soft-tissue lesions or inflammation, open wounds, pregnancy, an intrauterine device, patients undergoing corticosteroid therapy, haemophilia or treatment with anticoagulant therapy, hypersensitivity of the skin or dermatological diseases in the trunk making it impossible to apply the techniques, rejection of manual contact, non-Spanish-speaking patients, and patients who had previously received any myofascial release treatments.

The interventions were applied by a trained physical therapist with more than 10 years’ experience in MFR techniques.

### Intervention

The MFR group received a myofascial release treatment consisting of four sessions, each one lasting 25 minutes (twice a week for two weeks).

**Figure 1:**
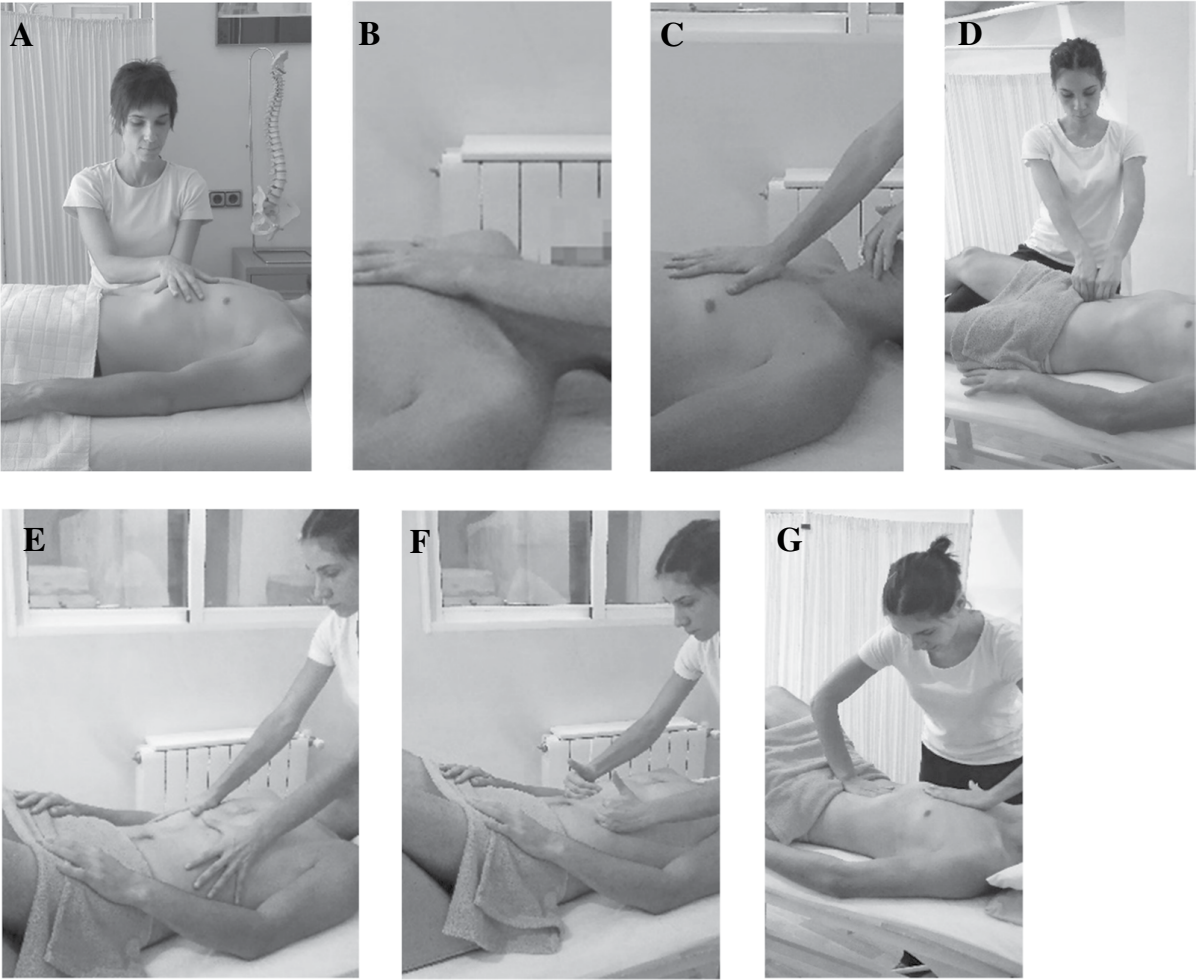
Myofascial release protocol for the intervention group. (**A**) Diaphragmatic transverse plane. (**B**) Anteroposterior equilibrium technique. (**C**) Supra and infrahyoid fascial induction. (**D**) Psoas fascial induction. (**E**) Diaphragm stretching technique: first step. (**F**) Diaphragm stretching technique: second step. (**G**) Phrenic centre inhibition.
B. Anteroposterior equilibrium technique: one contact hand was placed under the occiput and the other was placed at the sternum. A tridimensional distraction was applied between both hands, without sliding over the skin or forcing the tissue, for 3 minutes^16^ (Fig. 1B).
C. Supra and infrahyoid fascial induction (modified from Pilat *et al*.^9^): one hand was placed over the upper thoracic region and the other below the mandible. Tridimensional pressure was applied and the fascial movements were continued for 3 minutes (Fig. 1C).
D. Psoas fascial induction: the physical therapist put their knee under the patient’s knee on the side of the treatment. To confirm the correct placement of the psoas, the patient was asked to perform an isometric hip flexion. Both hands were then placed side by side with the finger tips in contact with the psoas. The physiotherapist performed three sets of 15 transverse slides at each psoas^9^ (Fig. 1D).
E. Diaphragm stretching technique: performed with the patient lying down and with flexed knees and a pillow under their head. This technique was performed in two steps:

Six MFR techniques were applied in each session:

A. Diaphragmatic transverse plane: bimanual transverse contact in the diaphragmatic region (T12–L1 and the xiphoid appendix), applying tridimensional pressure; the fascial movement was maintained for 5 minutes^9^ (Fig. 1A).

1. The physiotherapist placed both their hands at the lower ribs and exaggerated this movement while the patient took four deep breaths (Fig. 1E).
2. The physiotherapist put their hands under the lower ribs and while the patient took four deep breaths, accompanied the inspiratory movement while sustaining the rib gird to avoid its descension, stretching it laterally and in the cranial direction during the patient’s exhalation^13^ (Fig. 1F).

F. Phrenic centre inhibition: performed with the patient lying down with flexed knees and a pillow under their head. The therapist placed one hand over the sternum and the other on the abdomen. While the patient was exhalating the abdominal hand was moved in a cranial direction trying to go under the xiphoid appendix while the other hand pushed the sternum in a caudal direction. During the inspiration, all the forces were loosened to allow expansion of the thorax. This was done for 12 respiratory maneuvers^17^ (Fig. 1G).

The sham group received exactly the same type of contact treatment, following the respiratory movements but without applying any fascial stimulus or pressure, on the same schedule as the MFR group. To make participant blinding possible, all the patients were told that MFR therapy is a very gentle technique with very soft contact that is coordinated with the patient’s respiration, and patients who had previously experienced MFR therapy were excluded^10,18^.

### Outcome measures

#### Primary outcome

The primary outcome was self-reported changes in GERD symptoms, measured by the Reflux Disease Questionnaire (RDQ). This is a self-administered questionnaire comprising 12 items which measure the frequency and severity of heartburn, regurgitation, and dyspeptic complaints experienced by the patient in the prior week. It is scored on a Likert-type scale of 6 points ranging from ‘not present’ to ‘daily’ for the frequency and from ‘not present’ to ‘severe’ for the severity. Each participant’s score is the mean of item responses and ranges from 0 to 5, with higher scores indicating more frequent or severe symptoms. The RDQ is considered a good instrument for assessing responses to treatments in clinical trials^19^ and its validated translation to Spanish by Nuevo *et al*. (2009) showed high reliability and validity^20^.

#### Secondary outcomes

The secondary outcomes of this study were changes in health-related quality of life and PPIs use. The Gastrointestinal Quality of Life Index (GIQLI) is a self-administered questionnaire that assesses health-related quality of life related to gastrointestinal disturbances. It comprises 36 items scored from 0 to 4 (worse to better) and the GIQLI score is the sum of all the score items^21,22^. To register the PPIs usage (omeprazole), a calendar was given to the patients and they were asked to write down which days they took PPIs on and the dosage consumed in milligrams^3^. The PPIs score was the sum of the dose (in milligrams) the patient had ingested over the prior 7 days.

All these variables where measured at baseline (Week 0), one week after the end of intervention (Week 1) and at four weeks follow up (Week 4).

### Data analysis

The desired sample size was calculated after undertaking a pilot study of 20 participants, which indicated an effect size of 0.26 for the primary outcome (RDQ); considering this, as well as an α value of 0.05 and a desired power of 80%, we used the G*Power (v.3.0.10) program^23^ to estimate that a sample size of 26 participants was required. Accounting for potential losses of 15%, we established the final sample size of 30 participants.

Thus, 30 patients were randomly assigned either to the MFR (n = 15) or sham group (n = 15) using random allocation software using a block sampling technique of 10 participants^24^. This allocation was concealed from all staff members involved in every stage of the study. Whereas the manual therapist that applied the interventions was not possible to be blinded to the allocated arm, outcome assessors and data analysts were kept blinded to the allocation.

Data analysis was performed using SPSS (v.18.0) statistical software for Windows (SPSS Inc., Chicago, IL). To compare the results obtained in the MFR group and the sham group before and after the intervention, two-way mixed analysis of variance (ANOVA) tests were performed. The within-group factor was time, in which three levels were defined for all the variables (Week 0, Week 1, and Week 4). The between-group factor was group and had two levels: MFR and sham. All the statistical analyses were performed considering the overall score and without distinguishing by dimensions. To analyse the effectiveness of the participant’s blinding we performed chi-squared tests. All the analyses were performed with an intention to treat, and the significance level of the study was *P* < 0.05.

### Ethical approval

This study was approved by the University CEU Cardenal Herrera Human Ethics Committee CEI14/017 and followed the ethical guidelines set out in the Declaration of Helsinki. All participants gave written informed consent before data collection began.

## Results

### Flow of participants, therapists, and centres through the study

**Figure 2:**
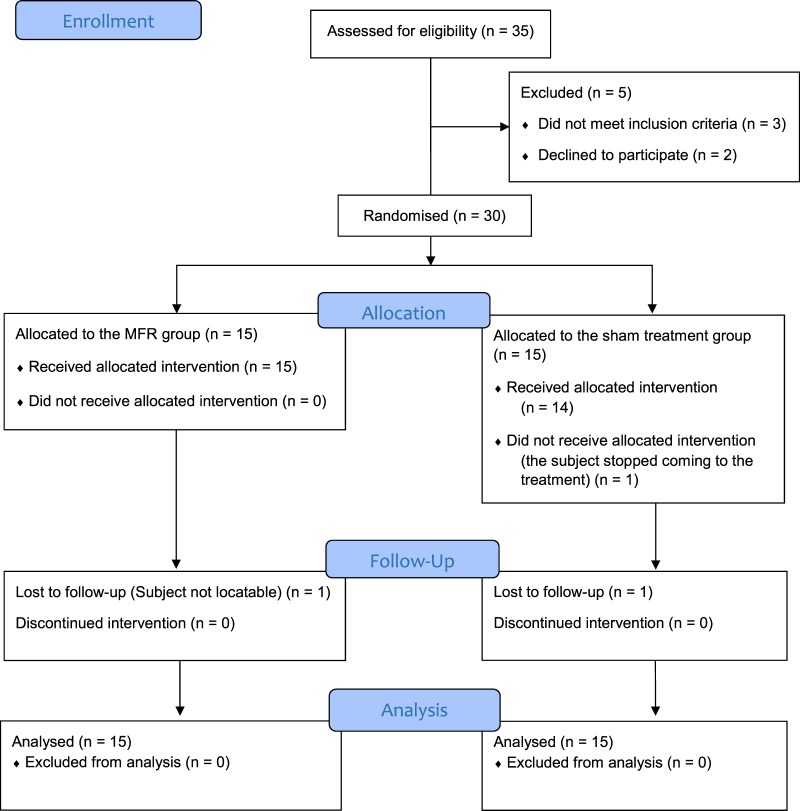
Progression of the participants through the trial.

We screened 35 consecutive participants in this clinical trial recruited from different medical centres of Valencia, of these, 2 were excluded because they had not previously undergone an endoscopy study, another participant was excluded because he was being treated for a duodenal tumour, and 2 more declined to participate. Table 1 shows the baseline characteristics of trial participants and Fig. 2 shows the progression of the participants through the trial.

**Table 1:** Participant characteristics at baseline.

	Group	
	MFR	Sham
Age	49.9 (14.4)	46.9 (14.8)
BMI (kg/m^2^)	25.6 (3.2)	27.3 (5.0)
Women/men	12/03	11/04
RDQ (0–5)	2.3 (1.0)	1.8 (1.0)
GIQLI (0–144)	102.3 (19.0)	94.3 (20.9)
PPI (mg/week)	88 (78)	135 (120)

MFR = Myofascial release group, Sham = sham group, BMI = body mass index; RDQ = Reflux Disease questionnaire; GIQLI = Gastrointestinal Quality of Life Index, PPI = Proton Pump Inhibitors.

Data are represented as the mean ± the standard deviation (SD).

### Compliance with trial method

The chi-squared test analysis showed no differences between the groups (p = 0.622), indicating that good participant blinding was achieved.

None of the patients reported any adverse event or intolerance to the intervention applied.

### Effects of interventions

The between-group analysis at week 4 highlighted a significant improvement in symptomatology (mean difference −1.1; 95% CI: −1.7 to −0.5), gastrointestinal quality of life (mean difference 18.1; 95% CI: 4.8 to 31.5), and PPIs use (mean difference −97 mg; 95% CI: −162 to −32) in the MFR group compared to the sham group (Table 2).

**Table 2:** Mean (SD) of each group, mean (SD) difference within each group, and mean (95% CI) difference between groups.

Outcome	Groups						Differences Within Groups				Difference Between Groups		
	Week 0		Week 1		Week 4		Week 1 minus Week 0		Week 4 minus Week 0		MFR minus Sham		
	MFR	Sham	MFR	Sham	MFR	Sham	MFR	Sham	MFR	Sham	Week 0	Week 1	Week 4
RDQ (0–5)	2.3 (1.0)	1.8 (1.0)	0.6 (0.6)	1.6 (0.8)	0.6 (0.5)	1.7 (1.0)	−1.7 (0.7)	−0.2 (0.7)	−1.6 (0.8)	−0.1 (0.6)	0.5 (−0.2 to 1.2)	−1.0** (−1.5 to −0.5)	−1.1** (−1.7 to −0.5)
GIQLI (0–144)	102.3 (19.0)	94.3 (20.9)	112.7 (13.2)	103.3 (22.7)	116.3 (14.6)	98.1 (20.7)	10.3 (8.2)	9 (18.1)	13.9 (12.8)	3.8 (8.7)	8.0 (−7.0 to 23.0)	9.3 (−4.5 to 23.2)	18.1* (4.8 to 31.5)
PPI (mg/week)	88 (78)	135 (120)	36 (52)	132 (122)	33 (51)	131 (112)	−52 (49)	−3 (10)	−55 (55)	−4 (43)	−47 (−122 to 29)	−96* (−166 to −26)	−97* (−162 to −32)

MFR = myofascial release group, Sham = sham group, RDQ = Reflux Disease questionnaire; GIQLI = Gastrointestinal Quality of Life Index, PPI = Proton Pump Inhibitors.

P-values were obtained from two-way ANOVA tests: **p ≤ 0.001, *p ≤ 0.01.

## Discussion

To the best of our knowledge, this preliminary study is the first designed to analyse the effects of a MFR protocol targeting the diaphragm for use in patients with non-erosive GERD. According to the results from the subjective standardised RDQ questionnaire, the application of this MFR protocol significantly reduced the frequency and severity of GERD symptoms and also appeared to improve the quality of life of patients with non-erosive GERD and reduce their consumption of PPIs. This finding concurs with other studies that used an active exercise protocol to increase the strength of the diaphragm in patients with GERD and produced results very similar to our own^3,5,6^. The use of MFR techniques in other areas of the body has also been shown to reduce pain^10^ and reaction time^25^, and to improve sliding of musculofascial junctions^18^ and patient range of motion and strength^10,18,25,26^. All of these effects can promote ease of movement and improve contractile capacity and are achieved because MFR techniques are supposed to ease myofascial restrictions and realign collagen fibres, helping to reset the soft tissue proprioceptive mechanism^25^.

Da Silva *et al*. (2013) used oesophageal manometry to confirm that using an MFR technique to stretch the diaphragm (one of the techniques we applied in this present study), immediately increased the pressure exerted on the EGJ, although they did not observe whether this effect was maintained over time^13^. On the other hand, the application of this same technique has been shown to improve diaphragmatic mobility and inspiratory capacity in patients with obstructive pulmonary disease^27^, which indirectly may imply an improvement of the functionality of the diaphragm. The improvements we describe here, in terms of the frequency and intensity of GERD symptoms, may be because MFR increased the contractile and proprioceptive capacity of the diaphragm, thus improving the effect the diaphragm has on the EGJ and allowing it to better function as an anti-reflux barrier.

On the other hand, the oesophagus receives dual sensory innervation from spinal and vagal nerves and the MFR treatment may have also exerted some regulatory effect on oesophageal peripheral innervation. The spinal nerve is believed to be responsible for nociceptive sensation, whereas vagal afferences are responsible for the perception of oesophageal distension and may also be involved in pain modulation^28^. In addition, the CD is widely innervated by the vagus nerve^29^. The antinociception action of vagal afferents seems to be mediated by activation of the descending cervical propriospinal neurons which inhibit the ascending spinothalamic pathways that transmit pain^30^. Given that many patients with non-erosive GERD suffer from visceral hypersensitivity, it is possible that mechanical stimulation of the CD may stimulate vagal afferences, thus decreasing nociceptive information transmitted by the spinal innervation of the oesophagus and therefore also reducing the symptoms of non-erosive GERD.

MFR techniques manipulate myofascial tissues—which form a network throughout every bodily tissue; thus, these protocols consider the human body as a holistic and continuous whole^9,31^. MFR therefore involves every muscular, osseous, and visceral tissue and helps to create space in which nerves, blood, and lymphatic vessels have improved maneuverability^32^. Both the oesophagus and branches of the vagus nerves pass alongside the oesophageal hiatus, which itself is wrapped inside the phrenoesophageal membrane^33^. This membrane is a layer of connective tissue and elastin that forms a sliding joint between the oesophagus and the diaphragm and is critical for establishing the correct respiratory and sphincter-function dynamics in the diaphragm at the level of the EGJ^34^. In this area of the body, using ultrasound, Rocha *et al*. (2015) showed that MFR improves diaphragmatic mobility^27^. Related to visceral fascial mobility, other authors showed that applying MFR techniques in the cervical region improved the sliding of the organs (thyroid, larynx, and oesophagus) related to these fascial tissues^18^. Considering that optimal phrenoesophageal membrane slip is necessary for correct antireflux barrier function^34^, applying MFR treatment at this level may improve function of the CD–oesophagus sliding component.

Here we showed a significant reduction in the consumption of PPIs among patients treated with MFR but not in the sham group. Such a reduction could also result in cost savings for the national health system. Moreover, treatment with MFR techniques has no side effects and could become a therapeutic alternative or complement in patients requiring long-term PPIs use or who want or need to avoid PPI medications because of their possible side effects^3,4^.

Finally, this study has some limitations. Firstly, we did not use any objective means to analyse possible changes at the EGJ (e.g., pH-metry, impedianciometry, or oesophageal manometry). It was therefore impossible to assess if there were any changes in the refluxate or in the strength of the EGJ. Second, it is very difficult to standardise the application of clinical interventions involving manual therapies because the pressure and sensitivity parameters used are impossible to unify. In this sense, the use of manual therapy is itself a study limitation^10,35^. Third, the design of this study did not allow us to determine whether any reduction in the symptoms and PPI use are maintained in the long term. On the other hand, instead sample size was calculated with a pilot study, wide CI were observed for the data which may be due to the relatively small sample size.

Despite these limitations, this is the first study to analyse the effects a MFR protocol applied in patients with non-erosive GERD has on their symptoms, quality of life, and PPI consumption. In summary, the MFR protocol we applied led to a statistically significant reduction in the symptoms and PPI use in patients with non-erosive GERD, and also improved their quality of life up to four weeks after the intervention. Future research should analyse any specific changes induced by MFR by using objective metrics in studies with larger sample sizes which include patients with GERD of varying severity, and should follow them up for longer periods.

## Conclusion

These preliminary findings indicate that the application of the MFR protocol we used in this study decreased the symptoms and PPIs usage and increased the quality of life of patients with non-erosive GERD up to four weeks after the end of the treatment.

## Data Availability

Data available on request from the authors.

## References

[1] Vakil N, Van Zanten SV, Kahrilas P, Dent J, Jones R (2006). The montreal definition and classification of gastroesophageal reflux disease: A global evidence-based consensus. Am J Gastroenterol..

[2] El-Serag HB, Sweet S, Winchester CC, Dent J (2014). Update on the epidemiology of gastro-oesophageal reflux disease: a systematic review. Gut..

[3] Eherer AJ (2012). Positive effect of abdominal breathing exercise on gastroesophageal reflux disease: A randomized, controlled study. Am J Gastroenterol..

[4] Lazarus B (2016). Proton pump inhibitor use and the risk of chronic kidney disease. JAMA internal medicine..

[5] Nobre e Souza MA (2013). Inspiratory muscle training improves antireflux barrier in GERD patients. American Journal of Physiology: Gastrointestinal & Liver Physiology..

[6] Sun X (2015). Short-term and long-term effect of diaphragm biofeedback training in gastroesophageal reflux disease: An open-label, pilot, randomized trial. Diseases of the Esophagus..

[7] Carvalho de MC, Suesada M, Polisel F, de Sá CC, Navarro-Rodriguez T (2012). Respiratory physiotherapy can increase lower esophageal sphincter pressure in GERD patients. Respir Med..

[8] Mittal RK (1990). Current concepts of the antireflux barrier. Gastroenterol Clin North Am..

[9] Pilat, A. Terapias miofasciales: Inducción miofascial. 1ª edición ed. Madrid: (ed. McGRAW - HILL - interamericana de España) (Madrid, 2003).

[10] Arguisuelas MD, Lison JF, Sanchez-Zuriaga D, Martinez-Hurtado I, Domenech-Fernandez J (2017). Effects of myofascial release in nonspecific chronic low back pain: A randomized clinical trial. Spine..

[11] Ajimsha MS, Al-Mudahka N, Al-Madzhar J (2015). Effectiveness of myofascial release: Systematic review of randomized controlled trials. Journal of Bodywork & Movement Therapies..

[12] Kage V, Bindra R (2015). Effect of active release technique v/s myofascial release on subjects with plantar fasciitis: A randomized clinical trial. Physiotherapy..

[13] Da Silva RC (2013). Increase of lower esophageal sphincter pressure after osteopathic intervention on the diaphragm in patients with gastroesophageal reflux. Dis Esophagus..

[14] Schulz KF, Altman DG, Moher D (2010). CONSORT 2010 statement: Updated guidelines for reporting parallel group randomised trials. BMJ..

[15] Williams JR (2008). The declaration of Helsinki and public health. Bull World Health Organ..

[16] Paleotti, S. *Las fascias. el papel de los tejidos en la mecánica humana* (ed. Editorial Paidotribo) 286–287 (Barcelona, 2004).

[17] Ricard, F. *Tratado de osteopatía visceral y medicina interna tomo I: Sistema cardiorrespiratorio*. (Editorial Madica Panamericana) (Buenos Aires, Madrid, 2008).

[18] Tozzi P, Bongiorno D, Vitturini C (2011). Fascial release effects on patients with non-specific cervical or lumbar pain. J Bodywork Movement Ther..

[19] Shaw M (2008). The reflux disease questionnaire: A measure for assessment of treatment response in clinical trials. Health and quality of life outcomes..

[20] Nuevo J, Tafalla M, Zapardiel J (2009). Validación del cuestionario sobre alteraciones de reflujo (RDQ) y de la escala de impacto de la enfermedad por reflujo gastroesofágico (GIS) para población española. Gastroenterología y hepatología..

[21] Quintana J (2001). Traducción y validación del índice de calidad de vida gastrointestinal (GIQLI). Revista español de enfermedades digestivas..

[22] Eypasch E (1995). Gastrointestinal quality of life index: Development, validation and application of a new instrument. Br J Surg..

[23] Faul F, Erdfelder E, Lang A, Buchner A (2007). G* power 3: A flexible statistical power analysis program for the social, behavioral, and biomedical sciences. Behavior research methods..

[24] Saghaei M (2004). Random allocation software for parallel group randomized trials. BMC medical research methodology..

[25] Kuruma H (2013). Effects of myofascial release and stretching technique on range of motion and reaction time. Journal of Physical Therapy Science..

[26] Khuman PR (2013). Myofascial release technique in chronic lateral epicondylitis: A randomized controlled study. Int. J. Health Sci. Res..

[27] Rocha T (2015). The manual diaphragm release technique improves diaphragmatic mobility, inspiratory capacity and exercise capacity in people with chronic obstructive pulmonary disease: A randomised trial. Journal of physiotherapy..

[28] Fass R, Tougas G (2002). Functional heartburn: The stimulus, the pain, and the brain. Gut..

[29] Young RL, Page AJ, Cooper NJ, Frisby CL, Blackshaw LA (2010). Sensory and motor innervation of the crural diaphragm by the vagus nerves. Gastroenterology..

[30] Chandler MJ, Zhang J, Qin C, Foreman RD (2002). Spinal inhibitory effects of cardiopulmonary afferent inputs in monkeys: Neuronal processing in high cervical segments. J Neurophysiol..

[31] Kumka M, Bonar J (2012). Fascia: A morphological description and classification system based on a literature review. J Can Chiropr Assoc..

[32] Guimberteau JC, Delage JP, McGrouther DA, Wong JK (2010). The microvacuolar system: How connective tissue sliding works. J Hand Surg Eur Vol..

[33] Filho JJO, Filho BH, Reis FP, Feitosa VLC, Aragão JA (2012). Contribution towards the anatomy of the esophageal hiatus and its relationship with the presence of bundles of collagen fibers in its margins. Int.J.Morphol..

[34] Delattre J, Avisse C, Marcus C, Flament J (2000). Functional anatomy of the gastroesophageal junction. Surg Clin North Am..

[35] Kidd RF (2009). Why myofascial release will never be evidence-based. International Musculoskeletal Medicine..

